# BUILDING AN ONLINE TRAINING PROGRAM FOR SAVVY FACILITATORS

**DOI:** 10.1093/geroni/igad104.1016

**Published:** 2023-12-21

**Authors:** Kenneth Hepburn, Carey Wexler Sherman, John Hobday, Adarsh Char, Lai Reed

**Affiliations:** Emory University, Atlanta, Georgia, United States; Savvy Systems, LLC, Minneapolis, Minnesota, United States; Savvy Systems, LLC, Minneapolis, Minnesota, United States; Full Tilt Ahead, LLC, Norcross, Georgia, United States; Emory University, Atlanta, Georgia, United States

## Abstract

Scaling evidence-based interventions to implementing organizations not linked to the development source poses difficulties in maintaining program fidelity. Program facilitator training must ensure consistency of training approach and content material across organizations. A training program must be able to certify that trainees can demonstrate a detailed understanding of the basic curriculum intent, learning objectives, core principles, mechanism of action, and operational details of the intervention. This presentation will describe the development of an online program to train interventionists to facilitate the Savvy Caregiver Program (SCP), a multi-session, group-based psychoeducational intervention grounded in Social Cognitive Theory that helps family caregivers develop the knowledge, skills, and sense of competency and confidence (mastery) important for effective caregiving. In collaboration with the educational design team at the Emory School of Nursing, we developed a prototype, fully asynchronous professional continuing educational interventionist training program. In seven components, the program employed embedded video, text, interactive interfaces, and short self-correcting quizzes to teach core principles, essential facilitator skills, and the dynamics of each of the six SCP sessions. At the end of each segment, learners were required to provide reflections on segment content; they were required to attain a score of at least 80% in order to advance to the next segment (retries were allowed). Based on feedback from the 35 interventionists who participated in the prototype training program, we added additional video assets to further illustrate methods for facilitating the program, further developed the internal quizzes and improved course navigation mechanisms.

